# The Blockade of Store-Operated Calcium Channels Improves Decompression Sickness in Rats

**DOI:** 10.3389/fphys.2019.01616

**Published:** 2020-01-31

**Authors:** Shih-En Tang, Wen-I Liao, Shu-Yu Wu, Hsin-Ping Pao, Kun-Lun Huang, Shi-Jye Chu

**Affiliations:** ^1^Division of Pulmonary and Critical Care Medicine, Department of Internal Medicine, Tri-Service General Hospital, National Defense Medical Center, Taipei, Taiwan; ^2^Graduate Institute of Aerospace and Undersea Medicine, National Defense Medical Center, Taipei, Taiwan; ^3^Department of Emergency Medicine, Tri-Service General Hospital, National Defense Medical Center, Taipei, Taiwan; ^4^Department of Internal Medicine, Tri-Service General Hospital, National Defense Medical Center, Taipei, Taiwan

**Keywords:** store-operated calcium channels, decompression sickness, lung injury, air embolism, BTP2

## Abstract

**Background:**

Previous investigations reveal that BTP2, a store-operated calcium channel blocker, has protective and anti-inflammatory properties in multiple inflammatory diseases. This study investigates whether BTP2 can protect against decompression sickness (DCS) in a rat model.

**Methods:**

BTP2 (2 mg/kg) was administered to male Sprague–Dawley rats 30 min before subjecting them to hyperbaric pressure. Control rats were not treated. After decompression, signs of DCS were examined, and samples of bronchoalveolar lavage fluid and lung tissue were obtained for evaluation.

**Results:**

The incidence and mortality of DCS were decreased significantly in rats treated with BTP2 compared to those treated with dimethyl sulfoxide. BTP2 significantly attenuated DCS-induced lung edema, histological evidence of lung inflammation, necroptosis, and apoptosis, while it decreased levels of tumor necrosis factor alpha, interleukin-6, and cytokine-induced neutrophil chemoattractant-1 in bronchoalveolar lavage fluid. In addition, BTP2 reduced the expression of nuclear factor of activated T cells and early growth response protein 3 in lung tissue. BTP2 also significantly increased the levels of inhibitor kappa B alpha and suppressed the levels of nuclear factor kappa B in lung tissue.

**Conclusion:**

The results suggest that BTP2 may has potential as a prophylactic therapy to attenuate DCS-induced injury.

## Introduction

Ca^2+^ signaling is known as one of the most abundant signaling molecules in biological processes that regulate many functions, such as metabolism; protein phosphorylation and dephosphorylation; neurotransmission; cell proliferation, division, and differentiation; gene transcription; muscle excitation–contraction; and programmed cell death (Karlstad et al., 2012). Abnormal Ca^2+^ signaling associates with inflammation and numerous disorders, such as Huntington’s disease, Alzheimer’s disease, and congestive heart failure (Karlstad et al., 2012). Ca^2+^ depletion of the endoplasmic reticulum (ER) stores can result in the opening of the store-operated calcium channels (SOCC) expressed in plasma membrane, which leads to more sustained Ca^2+^ signals. SOCC involves the integrated actions of stromal interaction molecular 1 (STIM1) and Orai1. STIM1 is a Ca^2+^-binding protein that is localized in the ER and functions as a sensor of ER Ca^2+^ depletion. Plasma membrane protein Orai1 is a selective Ca^2+^ channel pore protein of SOCC (Prakriya, 2013; Tian et al., 2016). During the depletion of ER Ca^2+^, STIM1 directly binds to Orai1 and activate it to mediate store-operated calcium channel calcium entry (SOCE). Sustained Ca^2+^ entry through SOCE is necessary for the activation of immune cells, including neutrophils, macrophages, and T cells (Prakriya, 2013; Tian et al., 2016).

Dysregulation of SOCE has been implicated in several diseases, such as severe combined immunodeficiency, inflammatory bowel disease, multiple sclerosis, acute pancreatitis, arterial thrombus formation, and collagen-induced arthritis (Prakriya, 2013; Tian et al., 2016; Zhang et al., 2017c). It has also been shown that bistrifluoromethyl pyrazole derivative, *N*-{4-[3,5-bis(trifluoromethyl)-1H-pyrazol-1-yl] phenyl}-4-methyl-1,2,3-thiadiazole-5-carboxamide (BTP2), a potent of SOCC inhibitor, can stop SOCE and successfully suppress SOCE-mediated functions of immune cells (Tian et al., 2016). Previous investigations have also revealed that BTP2 can improve ovalbumin-induced bronchial asthma, autoimmune hemolytic anemia, immunoglobulin G immune complex-induced inflammation, and the expression of spinal proinflammatory cytokines [i.e., interleukin-1β and tumor necrosis factor-α (TNF-α)] in a neuropathic pain model, as well as graft-versus-host disease in an animal model (Ohga et al., 2008a, b; Sogkas et al., 2015, 2018; Qi et al., 2016). Furthermore, BTP2 has been reported to attenuate ischemia–reperfusion (I/R), lipopolysaccharides (LPS), and ventilator-induced lung injury in rats (Gandhirajan et al., 2013; Zhang et al., 2017c; Song et al., 2018).

Decompression sickness (DCS) is an important medical problem in recreational scuba diving and is considered to be caused by bubble formation in the blood or tissue after a rapid drop in ambient pressure (Vann et al., 2011). When the sum of dissolved gas pressures (oxygen, carbon dioxide, nitrogen, and helium) in the blood and tissues exceeds ambient pressure, bubbles form in the tissues and blood vessels, which may lead to the clinical syndrome of DCS. The decompression bubbles can cause mechanical, embolic, and biochemical effects. Clinical manifestations of DCS can be reflected by direct influences of extravascular decompression bubbles, including pain due to the mechanical distortion of tissues, as well as tissue ischemia due to the obstruction of blood flow (Vann et al., 2011).

Endothelial injury from decompression bubbles can impair the integrity of the vessels, induce endothelial activation, alter vascular permeability, and lead to capillary breakdown with plasma leakage into the extravascular space. Furthermore, decompression bubbles may interact with the pulmonary endothelium and initiate platelet activation and deposition, apoptosis, oxidative stress, and leucocyte-endothelial adhesion. In turn, these effects may lead to a series of inflammatory reactions and, ultimately, endothelial damage (Li et al., 2009; Peng et al., 2010).

Recently, Zhang et al. reported a close correlation between bubble formation and endothelial injury in a DCS rat model (Zhang et al., 2016; Zhang et al., 2017b). Studies *in vitro* showed that the contact of air bubble with endothelial cells leads to increases in intracellular Ca^2+^, impaired cell activity and function, and subsequently, cell damage (Kobayashi et al., 2011; Sobolewski et al., 2011). Therefore, Ca^2+^ dysregulation may contribute to DCS. Based on the beneficial effects of BTP2 in various diseases and the pathophysiology of DCS, this study investigates whether BTP2 can protect against DCS.

## Materials and Methods

### Decompression Sickness

The rats in this study were cared for according to the guidelines of the National Institutes of Health, and all experiments were performed with the permission of the Animal Review Committee of National Defense Medical Center. Only animals within a weight range of 350 ± 20 g were used, due to the impact of body weight on DCS risk. The rats were placed in an acrylic hyperbaric chamber, which was pressurized with air to 6 absolute atmospheres (ATA) for 60 min (Huang et al., 2003). The chamber was ventilated with compressed air at 15 L/min to maintain a low-CO_2_ environment. The chamber temperature was kept constant at 27°C. Next, the animals were decompressed at a rate of 2 ATA/min and then transferred into individual cages. A dedicated staff member who was blinded to the drug treatment monitored the DCS symptoms for 2 h. The rats were diagnosed with DCS when one or more of the following symptoms appeared: dragging of the hind limb(s), dyspnea, agitation and rolling, and death (Huang et al., 2003). After the 2 h observation period, the surviving DCS rats were killed, and bronchoalveolar lavage fluid (BALF) and the lungs were collected for evaluation.

### Study Protocol

**FIGURE 1:**
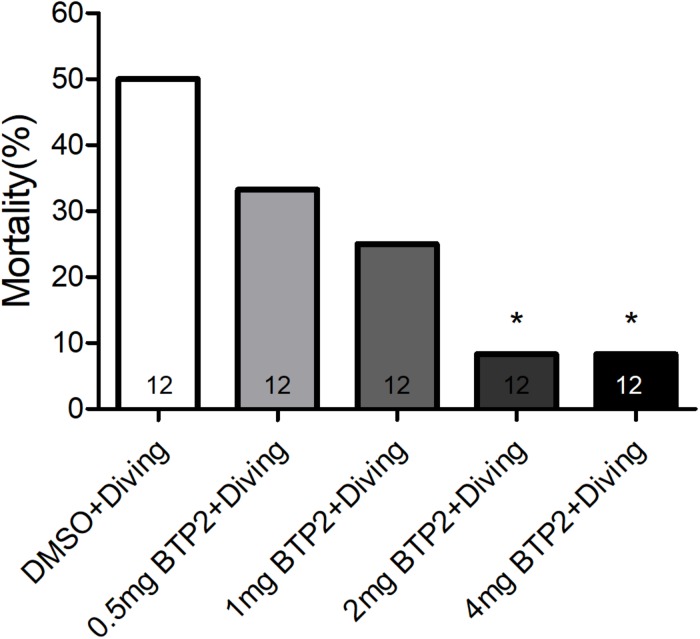
Effect of BTP-2 on the mortality of decompression sickness (DCS). Treatment with 0.5, 1.0, or 2.0 mg/kg BTP2 before decompression caused a dose-dependent decrease in the mortality of DCS, which reached significance at 2.0 mg/kg. Each group included 12 rats; *p < 0.05, compared with the DMSO + diving group.

BTP2 (Santa Cruz Biotechnology, CA, United States) was dissolved in dimethyl sulfoxide (DMSO) to create a stock solution. A total of 48 rats were randomly divided into one of four groups: the DMSO control group (*n* = 12), BTP2 control group (*n* = 12), DMSO + diving group (DMSO) (*n* = 12), and BTP2 + diving treatment group (BTP2) (*n* = 12). The control rats were exposed to normobaric condition in the hyperbaric chamber used for the diving simulation. The rats in the latter two groups were exposed to hyperbaric air and fast decompression to induce DCS. The doses of BTP2 (2 mg/kg) were chosen according to a pilot study, which showed that the administration of 2 mg/kg BTP2 before compression had maximal effects in decreasing the mortality ratio (Figure 1). The DMSO or BTP2 was administered 30 min before the compression procedure.

### Wet Lung to Dry Lung Weight Ratio

After the experiments, the right lung was removed from the hilar region. A part of the right lung lobe was weighed wet and incubated at 60°C for 48 h, and reweighed to obtain the dry weight. The wet/dry (W/D) weight ratio was determined by dividing the wet weight by the dry weight.

#### Measurement of Protein, Tumor Necrosis Factor-α, Cytokine-Induced Neutrophil Chemoattractant-1, and Interleukin-6 Levels in BALF

The left lung was lavaged twice with 2.5 ml of phosphate-buffered saline at the end of the experiment. The BALF was centrifuged at 200 × *g* for 10 min, and the protein concentration in the supernatant was determined using a bicinchoninic acid protein assay kit (Pierce, Rockford, IL, United States) according to the manufacturer’s instructions. The concentrations of TNF-α, interleukin-6 (IL-6), and cytokine-induced neutrophil chemoattractant-1 (CINC-1) in the BALF were measured using a commercial ELISA kit (R&D Systems Inc., Minneapolis, MN, United States) according to the manufacturer’s instructions.

### Histological Analyses

Paraffin sections of lung lobe were stained with hematoxylin–eosin (H&E) to evaluate the extent of lung injury. Semiquantitative grading of lung injury based on lung section histology was performed as previously described (Wu et al., 2015).

### Western Blot Analysis

The lung samples were separated by 10% sodium dodecyl sulfate polyacrylamide gel electrophoresis, and immunoblots were performed as described previously (Tang et al., 2014; Hung et al., 2019). The membranes were probed with anti-β-actin as a loading control (1:10,000, Sigma Chemical Company, St. Louis, MO, United States) and immunoblotted with antibodies against intracellular adhesion molecules (ICAM-1) (1:200, Santa Cruz Biotechnology, Dallas, TX, United States), nuclear factor of activated T-cells, cytoplasmic, calcineurin-dependent 1 (NFATc1) (1:500, Santa Cruz Biotechnology), early growth response protein 3 (Egr-3) (1:1,000, Santa Cruz Biotechnology), nuclear factor kappa B (NF-κB) p65, inhibitor of NF-κB (IκB)-α (1:1,000, Cell Signaling Technology, Danvers, MA, United States), proliferating cell nuclear antigen (1:1,000, Cell Signaling Technology, Danvers, MA, United States), and β-actin (1:10,000, Sigma Chemical Company, St. Louis, MO, United States). All data are presented as the ratio of the target protein to the reference protein.

### Immunohistochemical Detection

Immunohistochemical staining was performed to identify myeloperoxidase (MPO), receptor-interacting serine/threonine-protein kinase 3 (RIP3), and Egr-3 as described previously (Wu et al., 2015). Briefly, paraffin-embedded sections were deparaffinized in xylene before antigen retrieval. Endogenous peroxidase was then quenched with 3% H_2_O_2_ and 100% methanol for 15 min, and the lung sections were immunostained using rabbit anti-MPO (1:200, Thermo Fisher Scientific, Rockford, IL, United States), anti-RIP3 antibody (anti-RIP3, 1:200, ProSci, Fort Collins, CO, United States), and anti-Egr3 (anti-Egr3, 1:200, Santa Cruz Biotechnology). The slides were washed and then incubated with the secondary rat-specific horseradish peroxidase polymer antirabbit antibody (Nichirei Corporation, Tokyo, Japan) for 30 min. Next, horseradish peroxidase substrate was added and incubated for 3 min, and hematoxylin was used for counterstaining. A pathologist, who was blinded to the knowledge of the experiment groups, counted the MPO positive polymorphonucleated cells in six random fields per slide (×20). The average value of MPO-positive cells was computed from six animals per experiment group.

### TUNEL Assay of Lung Tissue

A terminal deoxynucleotidyl transferase dUTP nick end labeling (TUNEL) assay was performed using 5-mm-thick sections of paraffin-embedded lung tissue using a FragEL^TM^ DNA Fragmentation Detection Kit and Fluorescent-TdT Enzyme (Merk Millipore, Darmstadt, Germany) according to the manufacturer’s protocol. The TUNEL-positive nuclei were detected by fluorescence microscopy (Tang et al., 2014). The TUNEL-positive cells were quantified from 10 optical fields (×400) randomly chosen from each slide (*n* = 6 animals/group).

### Data Analysis

Statistical calculations for all data were performed using the statistical software GraphPad Prism 5 (GraphPad Software, San Diego, CA, United States). Groups were compared using a one-way ANOVA. Upon finding significant differences in the ANOVA, a Bonferroni *post hoc* test was used to examine relevant parameters. The incidence and mortality of DCS after decompression were compared using the chi-squared test. Data are presented as means ± SD, and values of *p* < 0.05 were considered significant.

## Results

### BTP2 Reduces the Incidence and Mortality of DCS

After rapid decompression, the mortality rate of DCS rats during 2 h of observation was 33%. BTP2 treatment significantly reduced the mortality of DCS (Table 1). As shown in Table 1, the incidence of DCS symptoms in the BTP2 group was significantly lower than in the DMSO group.

**TABLE 1:** Occurrence of signs of DCS in rats after rapid decompression from hyperbaric exposure of 6 ATA for 1 h.

	Without BTP2	BTP2 Pretreatment
	(*n* = 12)	(*n* = 12)
Signs of DCS	No. of rats	No. of rats
Dyspnea*	9 (75%)	3 (25%)
Death*	6 (50%)	1 (8%)
Dragging of hindlimbs	3 (25%)	1 (8%)
Agitation and rolling	1 (8%)	0 (0%)
Total*	9 (75%)	3 (25%)

Rats presenting ≥1 sign of DCS; *p < 0.05 Compared with the DMSO-DCS group.

### BTP2 Improves Pulmonary Microvascular Barrier Function

**FIGURE 2:**
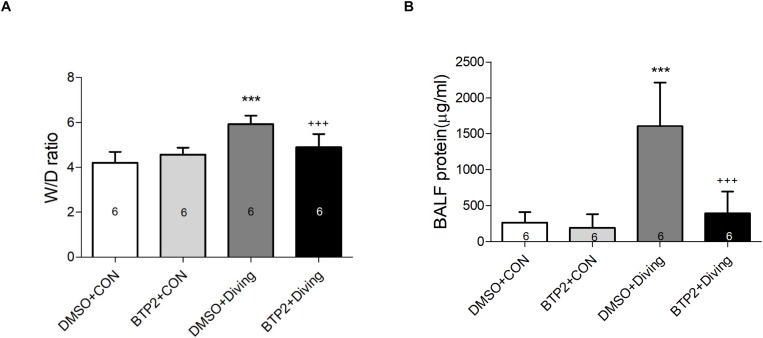
Effect of BTP-2 on acute lung injury induced by rapid decompression after hyperbaric exposure (6 atmospheres absolute) for 1 h. Lung wet weight/dry weight (W/D) ratio **(A)** and protein concentration in bronchoalveolar lavage fluid (BALF) **(B)** were measured after 2 h of observation. Data are expressed as means ± SDs (six rats per group); ***p < 0.001, compared with the DMSO + control group; ^+++^*p* < 0.001, compared with the DMSO + diving group.

Pulmonary edema was assessed by examining the lung W/D weight ratio and BALF protein concentration. As shown in Figures 2A,B, DCS significantly increased the lung W/D weight ratios and protein concentrations in the BALF after 2 h of observation (*p* < 0.001). However, treatment with BTP2 (2 mg/kg) significantly reduced these increases.

### BTP2 Attenuates the Expression of ICAM-1 Protein in the Lung Tissue and Proinflammatory Cytokines TNF-α, IL-6, and CINC-1 Levels in BALF

**FIGURE 3:**
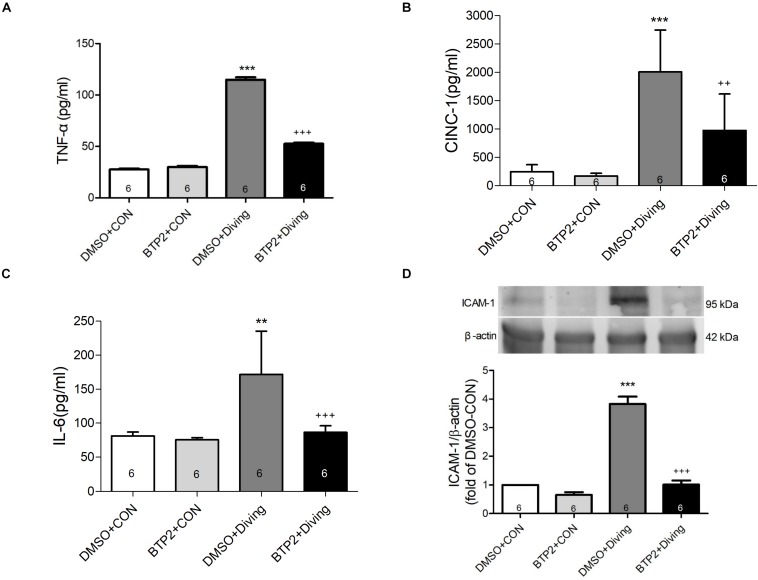
Effect of BTP-2 on TNF-α, CINC-1, and IL-6 levels in bronchoalveolar lavage fluid (BALF) and the expression of ICAM-1 protein in lung tissue. TNF-α **(A)**, CINC-1 **(B)**, and IL-6 **(C)** were measured after 2 h of observation. Western blot analysis of ICAM-1 **(D)** protein in the lung tissue. β-actin served as a loading control for cytoplasmic proteins. Representative blot is shown. Data are expressed as mean ± SDs (six rats per group). **p < 0.01, ***p < 0.001, compared with the DMSO + control group; ^++^*p* < 0.01, ^+++^*p* < 0.001, compared with the DMSO + diving group.

Decompression sickness led to significantly increased levels of TNF-α, IL-6, and CINC-1 in the BALF (*p* < 0.01–*p* < 0.001; Figures 3A–C) and the expression of ICAM-1 in the lung tissue after 2 h observation (*p* < 0.001; Figure 3D). BTP2 (2 mg/kg) significantly suppressed these effects.

### BTP2 Improves Lung Histopathology in DCS Rats

**FIGURE 4:**
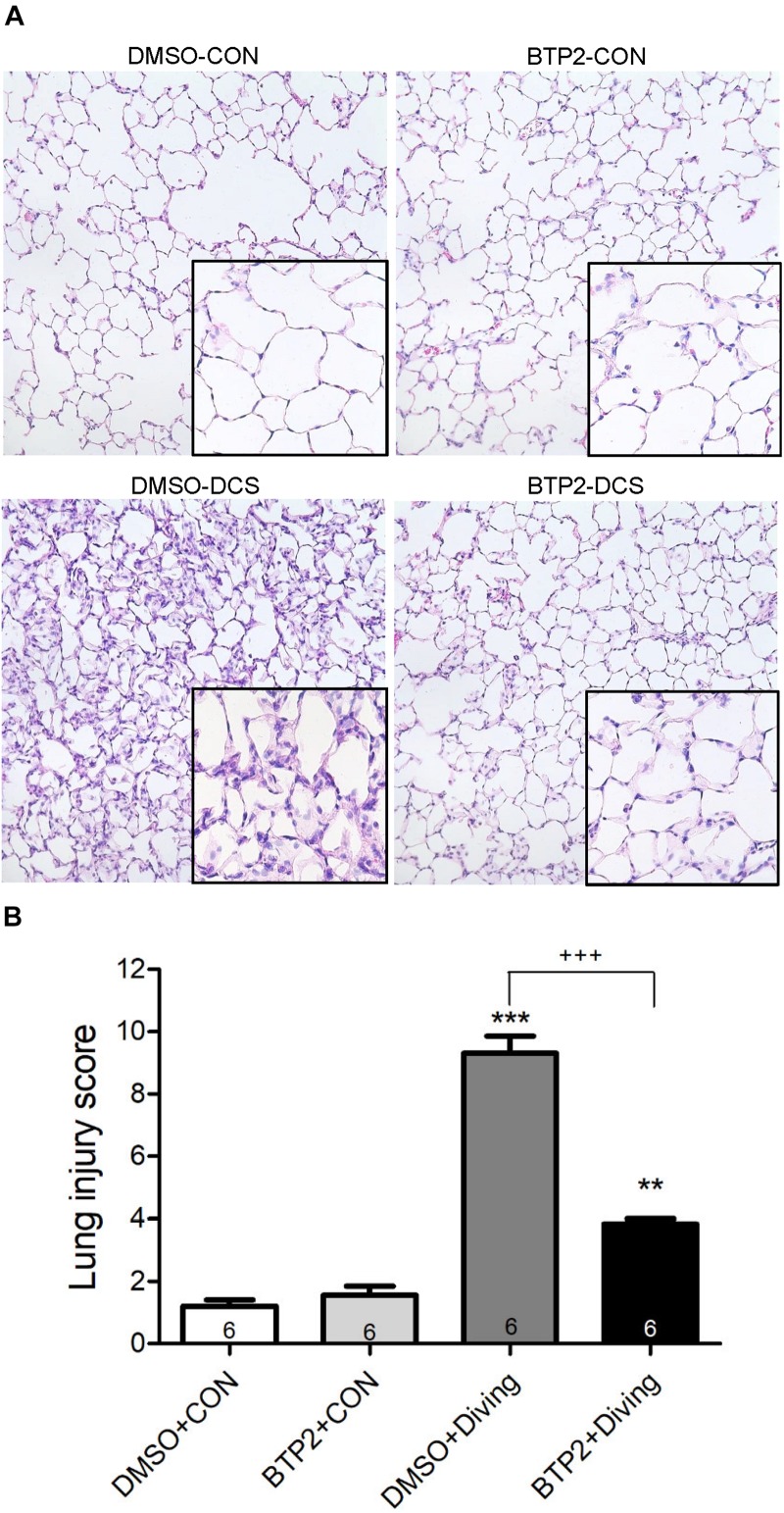
Effect of BTP-2 on lung histopathology. Representative micrograph of lung tissue (representative results, 200×, hematoxylin and eosin staining). **(A)** Neutrophil infiltration and septal edema were increased in the DMSO + diving group. BTP-2 treatment significantly attenuated these histopathological changes and the lung injury scores. **(B)** Data are expressed as mean ± SDs (six rats per group). **p < 0.01, ***p < 0.001, compared with the DMSO + control group; ^+++^*p* < 0.001, compared with the DMSO + diving group.

**FIGURE 5:**
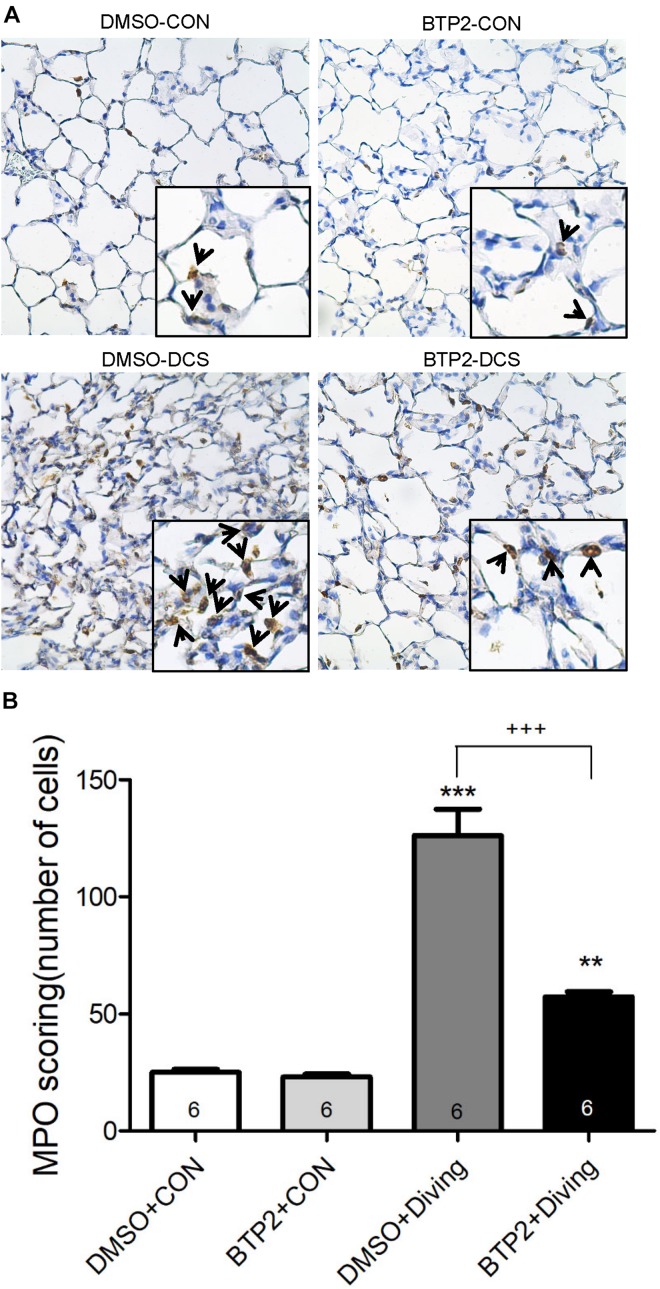
Effect of BTP-2 on MPO-positive cells in lung tissue. Immunohistochemical analysis (200×) of MPO **(A)** and number of MPO-positive cells quantified **(B)**. BTP-2 treatment significantly decreased the number of MPO positive cells **(B)**. **p < 0.01, ***p < 0.001, compared with the DMSO + control group; ^+++^*p* < 0.001, compared with the DMSO + diving group.

Decompression sickness led to histological abnormalities in lung tissues, including the infiltration of leukocytes and a marked thickening of the interalveolar walls. These alterations were attenuated in DCS rats that received BTP2 (2 mg/kg) (Figure 4A). Treatment with BTP2 (2 mg/kg) also significantly reduced lung injury scores (Figure 4B). The DCS group had significantly increased levels of MPO-positive cells in lung tissue compared with the DMSO + control group (Figure 5). Treatment with BTP2 (2 mg/kg) significantly attenuated the observed increases.

### BTP2 Attenuates Apoptosis in Lung Tissue of DCS Rats

**FIGURE 6:**
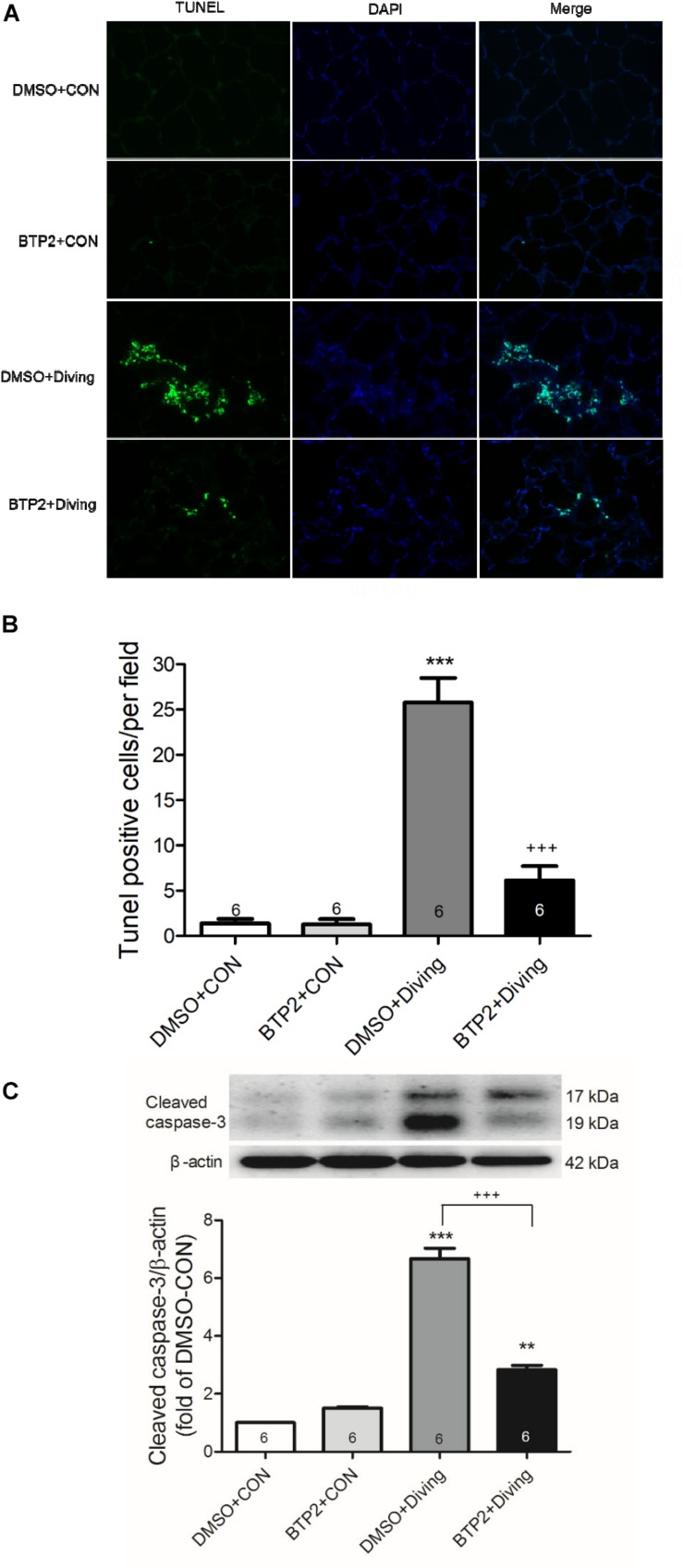
Effect of BTP-2 on expression of caspase-3 and DNA fragmentation in lung tissue. TUNEL assay of lung tissue **(A)**. Counting the TUNEL-positive cells from 10 randomly chosen fields **(B)**. Western blot analysis of caspase-3 **(C)** protein in lung tissue. β-actin served as a loading control for cytoplasmic proteins. Representative blot is shown. DCS significantly increased DNA fragmentation and caspase-3 expression in the lung tissue. BTP-2 treatment significantly attenuated these increases. Data are expressed as mean ± SDs (six rats per group). **p < 0.01, ***p < 0.001, compared with the DMSO + control group; ^+++^*p* < 0.001, compared with the DMSO + diving group.

The number of TUNEL-positive cells (Figures 6A,B) and the protein levels of cleaved caspase-3 (Figure 6C) significantly increased in the lungs of DCS rats. BTP2 pretreatment significantly reduced the amount of apoptosis.

### BTP2 Suppresses Necroptosis in Lung Tissue of DCS Rats

**FIGURE 7:**
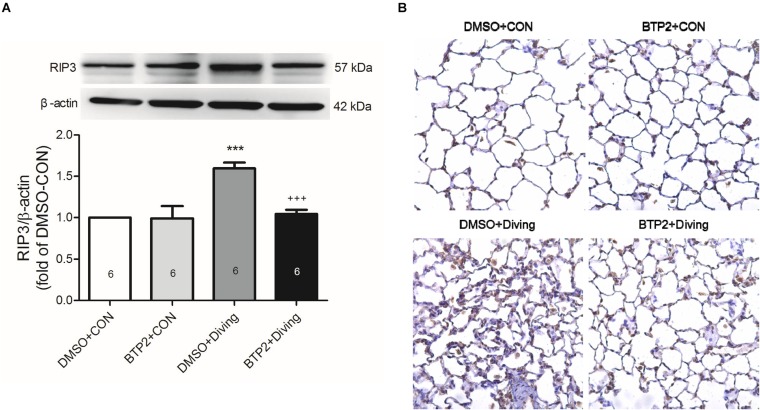
Effect of BTP-2 on the expression of RIP3 in lung tissue. Western blot analysis of RIP3. **(A)** Immunohistochemical analysis (200×) of RIP3. **(B)** β-actin served as a loading control for cytoplasmic proteins. Representative blot is shown. DCS significantly increased RIP3 protein expression in the lung tissue. BTP-2 treatment significantly decreased these increases. Data are expressed as mean ± SDs (six rats per group). ***p < 0.001, compared with the DMSO + control group; ^+++^*p* < 0.001, compared with the DMSO + diving group.

Immunohistochemical and Western blot analyses of lung tissue indicated that DCS significantly increased the expression of RIP3 (*p* < 0.05, Figure 7), which is consistent with increased necroptosis. Treatment with BTP2 (2 mg/kg) significantly reversed the effect.

### BTP2 Suppresses NFATc1 and Egr-3 Expression in the Lung Tissue of DCS Rats

**FIGURE 8:**
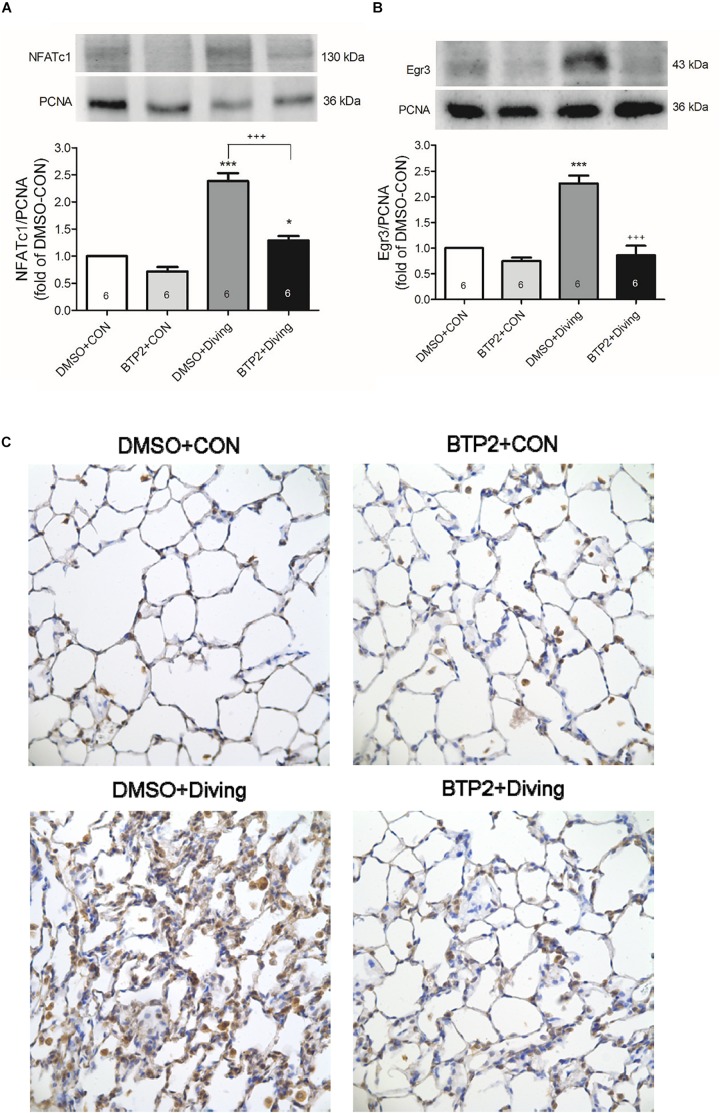
Effect of BTP-2 on NFATc1 and Egr-3 expression in lung tissue. Western blotting was used to measure the NFATc1 **(A)** and Egr-3 **(B)**. Immunohistochemical analysis (200×) of Egr-3 **(C)**. Equal loading was confirmed with proliferating cell nuclear antigen (PCNA). Representative blots are shown. Data are expressed as mean ± SDs (six rats per group). *p < 0.05, ***p < 0.001, compared with the DMSO + control group; ^+++^*p* < 0.001, compared with the DMSO + diving group.

Western blot analysis of the lung tissue indicated that DCS significantly increased the NFATc1 translocation and Egr-3 expression in the lung tissue after 2 h of observation (Figure 8). Immunohistochemical analysis of the lung tissue showed that DCS significantly increased the Egr-3 expression. Treatment with BTP2 (2 mg/kg) significantly suppressed all of these effects.

### BTP2 Inhibits Activation of NF-κB Pathway in the Lung Tissue of DCS Rats

**FIGURE 9:**
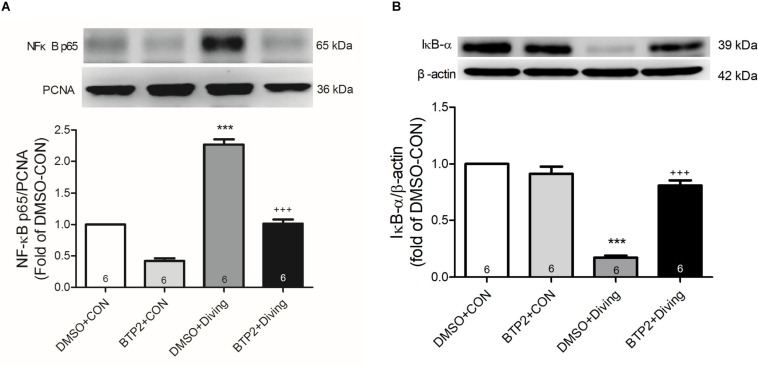
Effect of BTP-2 on NF-κB activation in lung tissue. Western blotting was used to measure nuclear NF-κB p65 **(A)** and cytoplasmic IκB-α **(B)**. Proliferating cell nuclear antigen (PCNA) and β-actin served as loading controls for nuclear and cytoplasmic proteins, respectively. Representative blots are shown. Data are expressed as mean ± SDs (three rats per group). ***p < 0.001, compared with the DMSO + control group; ^+++^*p* < 0.001, compared with the DMSO + diving group.

Western blot analysis of the lung tissue indicated that DCS led to a significantly increased nuclear level of NF-κB p65 and a significantly decreased level of cytoplasmic IκB-α after 2 h of observation (Figure 9). Treatment with BTP2 (2 mg/kg) significantly suppressed these effects.

## Discussion

Our results showed that BTP2 treatment had a dose-dependent therapeutic effect on the mortality of rats subjected to DCS. Furthermore, it significantly reduced the incidence of DCS and attenuated DCS-induced lung injury, which was evidenced by the reduction in lung W/D ratios and protein levels in BALF. BTP2 treatment also suppressed the DCS-induced production of proinflammatory cytokines in BALF, the influx of pulmonary neutrophils, and tissue damage. At the molecular level, BTP2 treatment inhibited DCS-induced necroptosis and apoptosis, as indicated by decreased RIP3 expression, protein levels of cleaved caspase-3, and TUNEL-positive cells in the lungs. Furthermore, BTP2 treatment also attenuated NFAT, Egr-3, and NF-κB expression in the lung tissue and reduced DCS-induced inflammation. These results imply that BTP2 may have promise as an adjunct treatment for DCS and may be considered for future clinical studies.

Alterations in the amplitude of cytosolic Ca^2+^ levels have long been recognized as a critical regulator of cell function (Karlstad et al., 2012). Plasma membrane SOCCs are channels that can be turned on by depleting ER Ca^2+^ stores. In addition, the opening of SOCCs replenishes the Ca^2+^ stores of the ER. SOCCs have been linked as important actors to immune and inflammatory diseases (Prakriya, 2013). During DCS injury, air bubbles that contact endothelial cells induce an increase in intracellular calcium that is released from the intracellular calcium stores and via pathological calcium influx through the plasma membrane. This elevated cytosolic calcium causes cellular damage, increases endothelial cell permeability, and allows leakage of protein-rich edema fluid into the alveolus, which impairs gas exchange and induces hypoxemia. BTP2 treatment inhibits calcium flux through SOCCs and subsequently decreases endothelial injury. This result is consistent with previous studies demonstrating that BTP2 can attenuate the increased endothelial permeability in I/R, LPS, and ventilator-induced lung injury (Gandhirajan et al., 2013; Zhang et al., 2017c; Song et al., 2018).

A number of experiments have shown that gas bubble injuries induce the rapid release of proinflammatory cytokines (Li et al., 2009; Peng et al., 2010; Zhang et al., 2017a; Mazur et al., 2018). It is possible that air bubbles trigger the activation of alveolar macrophages, which then release many mediators (e.g., TNF-α, CINC-1, and IL-6). In this study, DCS increased the levels of proinflammatory cytokines TNF-α, IL-6, and CINC-1 in the BALF and the expression of ICAM-1 protein, which were decreased by the administration of BTP2. This indicates that BTP2 attenuated local and systemic inflammation and may contribute to improving lung injuries. The results are comparable with those of previous studies demonstrating that BTP2 can attenuate inflammatory cytokine production (Gandhirajan et al., 2013; Zhang et al., 2017c).

The term necrosis describes cell death with the swelling of organelles and plasma membrane rupture. Programmed necrosis is induced by many stimuli, such as intracellular ATP depletion, disturbance of Ca^2+^ homeostasis, mitochondrial depolarization, poly-(ADP-ribose) polymerase activation, proteolysis by non-apoptotic proteases, increased reactive oxygen species, and cell surface receptor activation. Necroptosis has been demonstrated in several animal models of diseases, such as renal and myocardial I/R injury, acute pancreatitis, skin and intestinal inflammation, LPS-induced lung injury, and TNF-induced systemic inflammatory response syndrome (Duprez et al., 2011; Linkermann and Green, 2014).

Receptor-interacting serine/threonine-protein kinase 3 was identified as a crucial regulator of death receptor-induced necrosis. The most extensively characterized pathway leading to RIP3 activation during necrosis is initiated by TNF-α. In a previous study, RIP3-knockout mice were protected from LPS-induced lung injury and TNF-α-induced systemic inflammatory response syndrome (Duprez et al., 2011; Wang et al., 2016). In our study, we found that the levels of RIP3 in lung tissues and TNF-α in BALF increased after DCS challenge and were significantly downregulated by the administration of BTP2. It is very likely that the elevation of RIP3 expression occurs as a consequence of tissue damage. The results indicated that BTP2 improved lung injury, possibly via a RIP3-dependent necroptosis pathway. Previous studies have revealed that apoptosis involved in the process of decompression injury after simulated diving in rats and has been implicated as an important pathophysiological process in DCS (Ni et al., 2013). In the current study, the level of the proapoptotic cleaved-caspase-3 and the number of TUNEL-positive cells significantly increased in DCS rats, and BTP2 provided significant protection against the injuries. Therefore, both apoptosis and necroptosis contributed to cell death in DCS. However, the crosstalk between the regulatory pathways of apoptosis and necroptosis in DCS needs further examination (Vanden Berghe et al., 2015).

Ca^2+^ oscillations are known to control cellular responses, including gene expression, by differentially triggering transcription factors such as NFAT and NF-κB (Fisher et al., 2006). NFAT proteins are thought to be important controllers of the production of cytokines such as TNF-α (Fric et al., 2012). The nuclear translocation and transcriptional activation of NFAT is primarily driven by Ca^2+^ oscillations mediated by STIM/Orai channels (Fric et al., 2012). Stim1 knockout in endothelial cells led to a significant decrease in the nuclear accumulation of NFAT, the expression of NFAT-driven cytokines, and NFAT luciferase activity after LPS stimulation (Gandhirajan et al., 2013). Furthermore, the Ca^2+^ blocker BTP2 inhibited NFAT translocation upon LPS exposure. These findings suggest that STIM1-mediated Ca^2+^ influx is required for the LPS-induced NFAT activation and the production of inflammatory cytokines (Gandhirajan et al., 2013). Egr3 is a zinc-finger transcription factor in the Egr gene family, which consists of four isoforms: Egr1, 2, 3, and 4. Endothelial cells demonstrated rapid and profound induction of Egr3 in response to a wide range of stimuli, such as activation and differentiation signals, TNF-α, and tissue injury (Suehiro et al., 2010). Furthermore, NFAT binds to the promoter regions of the Egr3 gene to transcriptionally enhance the expression of Egr3, which is involved in leukocyte trafficking, the expression of chemokines (e.g., CXCL1, IL-8), and vascular permeability (Suehiro et al., 2010). In this study, BTP2 suppressed the activation of NFAT and thus inhibited the Egr3 gene expression in the lung tissue of DSC rats, thereby preventing NFAT-regulated cytokine expression. Our findings are comparable with those of a previous study (Gandhirajan et al., 2013), suggesting that the inhibition of NFAT–Egr-3 interaction by BTP2 may at least in part suppress the inflammation induced by DCS.

NF-κB is an important a protein complex that is involved in the expression of many proinflammatory cytokines and chemokines to stimuli, such as stress and cytokines. It also results in various inflammatory conditions. Cytoplasmic NF-κB activation involves IκB phosphorylation by IκB kinase, followed by translocation to the nucleus, where NF-κB binds to the promoter regions of various genes and prompts the production of proinflammatory cytokines (Wu et al., 2015; Liao et al., 2017). Previous studies have suggested that NF-κB activation plays a role in the pathogenesis of air embolism-induced lung injury (Li et al., 2009; Peng et al., 2010). Increasing intracellular Ca^2+^ signals is known to enhance the nuclear translocation of the NF-κB p65 protein and NF-κB-dependent transcription, which can lead to the production of proinflammatory mediators (Karlstad et al., 2012). Liu et al. (2016) showed that the influx of extracellular Ca^2+^ via STIM1 and Orai1 is critical for T-cell receptor induced IκB degradation and NF-κB activation. They showed that the Ca^2+^-dependent phosphatase calcineurin activated NF-κB *in vitro*, which increased Ca^2+^ levels induced by ionomycin-enhanced NF-κB activation. However, the removal of Ca^2+^ by a Ca^2+^ chelator attenuated both processes (Liu et al., 2016). We also observed similar results, as the inhibition of Ca^2+^ mobilization by BTP2 significantly inhibited IκB degradation and NF-κB activation. It also led to the attenuated production of proinflammatory cytokines, such as TNF-α and CINC-1 and reduced leukocytes infiltration. Thus, the anti-inflammatory action of BTP2 could be partly due to the inhibition of NF-κB signaling and the consequent production of proinflammatory cytokines.

The present work has some limitations. First, our study only investigated the efficacy of BTP2 as a preventive potential. Because the initiation of DCS is obscure and hard to observe clinically. When signs and symptoms of DCS can be detected, it may be difficult to stop the development of DCS. Whether BTP2 has therapeutic benefit when DCS is already developed is not clear. Further research is needed to elucidate the therapeutic potential of BTP2.

The results of our study indicate that the SOCE inhibitor BTP2 provides protective effects in rats subjected to DCS injury. Furthermore, BTP2 appears to reduce necroptosis and apoptosis, suppress the activation of NFAT and NF-κB, and thereby inhibit the production of proinflammatory mediators and cytokine. These effects are essential for the mounting of an inflammatory response to DCS. Therefore, our study provides evidence of the key role of SOCE in DCS injury and indicates that SOCE inhibitors may be a potential adjunct therapy for DCS. Nevertheless, a better understanding of the physiological action of SOCE in DCS is needed before it can be suggested for clinical application.

## Data Availability Statement

The data gathered in this study are available upon request to the corresponding author.

## Ethics Statement

The animal study was reviewed and approved by the Animal Review Committee of National Defense Medical Center.

## Author Contributions

S-ET conceived and designed the experiments, and performed the experiments. W-IL, H-PP, and S-YW analyzed the data. S-JC and K-LH wrote the manuscript. All authors read and approved the final manuscript.

## Conflict of Interest

The authors declare that the research was conducted in the absence of any commercial or financial relationships that could be construed as a potential conflict of interest.
